# Fidelity considerations in translational research: Eating As Treatment — a stepped wedge, randomised controlled trial of a dietitian delivered behaviour change counselling intervention for head and neck cancer patients undergoing radiotherapy

**DOI:** 10.1186/s13063-015-0978-5

**Published:** 2015-10-15

**Authors:** Alison Kate Beck, Amanda Baker, Ben Britton, Chris Wratten, Judith Bauer, Luke Wolfenden, Gregory Carter

**Affiliations:** Centre for Translational Neuroscience and Mental Health, University of Newcastle, Callaghan, NSW 2308 Australia; Department of Radiation Oncology, Calvary Mater Newcastle Hospital, Waratah, NSW 2308 Australia; Centre for Dietetics Research, The University of Queensland, St Lucia, QLD 4072 Australia; School of Medicine & Public Health, The University of Newcastle, Callaghan, NSW 2308 Australia

**Keywords:** Treatment fidelity, Treatment Integrity, Behaviour change counselling, Implementation, Head and neck cancer

## Abstract

**Background:**

The confidence with which researchers can comment on intervention efficacy relies on evaluation and consideration of intervention fidelity. Accordingly, there have been calls to increase the transparency with which fidelity methodology is reported. Despite this, consideration and/or reporting of fidelity methods remains poor. We seek to address this gap by describing the methodology for promoting and facilitating the evaluation of intervention fidelity in The EAT (Eating As Treatment) project: a multi-site stepped wedge randomised controlled trial of a dietitian delivered behaviour change counselling intervention to improve nutrition (primary outcome) in head and neck cancer patients undergoing radiotherapy.

**Methods/Design:**

In accordance with recommendations from the National Institutes of Health Behaviour Change Consortium Treatment Fidelity Workgroup, we sought to maximise fidelity in this stepped wedge randomised controlled trial via strategies implemented from study design through to provider training, intervention delivery and receipt. As the EAT intervention is designed to be incorporated into standard dietetic consultations, we also address unique challenges for translational research.

**Discussion:**

We offer a strong model for improving the quality of translational findings via real world application of National Institutes of Health Behaviour Change Consortium recommendations. Greater transparency in the reporting of behaviour change research is an important step in improving the progress and quality of behaviour change research.

**Trial registration number:**

ACTRN12613000320752 (Date of registration 21 March 2013)

## Background

### What is treatment fidelity?

Treatment fidelity encompasses strategies designed to monitor and enhance the reliability and validity of behavioural interventions [1]. Broadly, it encompasses integrity (whether an intervention was delivered as intended) and differentiation (the degree to which the intervention is distinguishable from other study arms) [1]. Thorough consideration of treatment fidelity improves the confidence with which conclusions can be drawn regarding treatment efficacy. The presence or absence of treatment effects can be more clearly linked to the intervention under investigation relative to non-adherence or non-specific factors. In practice, this means less risk of prematurely rejecting potentially effective treatments, and conversely, further evaluating and/or adopting ineffective treatments [2]. Moreover, there is some evidence to suggest that high-fidelity interventions generate superior outcomes compared to low-fidelity interventions [3].

### Components of treatment fidelity

In 2004 the National Institutes of Health (NIH) Behavioural Change Consortium developed best practice recommendations for assessing, monitoring and enhancing treatment fidelity within behaviour change interventions [2]. Within these recommendations, fidelity was conceptualised according to five domains: 1) study design, 2) training, 3) delivery, 4) receipt and 5) enactment (see Table 1; [2, 4]). More recently, following a synthesis of 30 years of literature, Gearing, El-Bassel, Ghesquiere, Baldwin, Gillies and Ngeow [5] derived a four-component model reflecting the first four domains described by Bellg et al. However, unlike Bellg et al. [2], Gearing et al. [5] conceptualised enactment (that is, whether the patient applies learned skills in his or her daily life) as a component of treatment efficacy rather than treatment fidelity and excluded it from their conceptualisation. That is, patients may remain unable or unwilling to apply skills and/or knowledge even though the clinician may be faithful to intervention delivery.

**Table 1:** NIH fidelity recommendations

Fidelity component	Aim	Key considerations
**Study design:** The precision with which the ‘active’ components of the intervention can be assessed	To facilitate adequate hypothesis testing regarding underlying theory and clinical processes via:	• Intervention theory, goal and strategies including structure and delivery, role of interventionists, topics, activities, equipment and materials, mode of delivery
	(1) Ensuring the intervention has sound theoretical underpinnings	• Treatment dose (for example, minimum and ideal frequency, duration and number of sessions)
	(2) Monitoring and minimising contamination within and between treatment arms	• Troubleshooting (for example, interventionist dropout)
	(3) Measuring treatment dose and intensity	
	(4) Identifying and addressing potential setbacks in intervention implementation	
**Training providers:** The consistency and adequacy of training	To ensure competent acquisition and maintenance of skills to equip providers to effectively deliver the intervention via	• Interventionist differences (for example, skill, education, experience and implementation style)
	(1) Standardisation	• Threats (for example, intervention complexity and drift in delivery over time)
	(2) Steps to minimise skill ‘decay’ or ‘drift’ over time	
**Delivery of treatment:** Whether the intervention was delivered as intended	To ensure that the intervention is delivered as intended via (1) Standardisation and monitoring	• Behaviours that are unique; essential, but not unique; compatible, but neither essential nor unique and prohibited
		• Skill with which the intervention is delivered
		• Non-specific treatment effects (for example, warmth, rapport)
		• Assessment method (for example, reliability and validity of assessment measures; assessors; training)
		• Threats (for example, mismatch between intervention and practitioner skill/education/self-efficacy; intervention complexity; contamination across treatment conditions)
**Receipt of treatment:** Whether the patient can understand and perform treatment-related behaviours	To monitor and improve patient capacity to acquire knowledge and skills	• Comprehension of, engagement in and adherence to intervention content
		• Dose received
**Patient enactment:** Whether the patient actually performs treatment-related skills in the real world	To monitor and improve patient application of knowledge and skills in real life settings	• NA

### Intervention fidelity and complex behaviour interventions

Consistent with the overall paucity of detail with which complex behaviour change interventions tend to be described [6], there is considerable variability in the reporting and/or conduct of intervention fidelity procedures. In a review of 342 health behaviour change interventions conducted between 1990 and 2000, 54 % of studies failed to report on intervention fidelity [1]. Furthermore, intervention delivery tends to be monitored and/or reported, to the relative neglect of other important fidelity components (including intervention design, training and receipt) [5]. Within the psycho-oncology literature this trend is replicated, with a recent review demonstrating that none of the 28 studies reviewed met criteria for ‘high intervention fidelity’ in their reporting, implementation and/or adherence to NIH fidelity strategies [7].

Inadequate consideration and reporting of fidelity procedures undermines our capacity to understand and replicate treatment effects and subsequently to disseminate effective complex behaviour change interventions. Informed by the NIH guidelines [2] and the recommendations offered in a subsequent review [5], we seek to bridge this gap in the literature by discussing the fidelity strategies adopted in our multi-site evaluation of a dietitian delivered behaviour change counselling (BCC) intervention. Specifically, we will discuss the strategies used to promote intervention fidelity, including the data collection methods and process variables to be used in future evaluations. Our protocol provides a working model for treatment fidelity implementation within multi-site translational evaluations of complex behaviour change interventions.

## Methods/Design

### EAT: radiotherapy nutrition project overview

This fidelity protocol is nested within a clinical trial that is currently underway. The Eating As Treatment (EAT) project is a multi-site stepped wedge randomised controlled trial evaluating the effectiveness of a BCC intervention relative to usual care for improving nutritional status amongst adult patients with head and neck cancer (HNC) undergoing radiotherapy (for study protocol, see [8]). A total of 400 patients will be recruited from radiotherapy departments in six Australian hospitals. As per the stepped wedge design [9], all sites began in the control phase. Following a randomly determined order, training was provided and sites commenced intervention delivery. ‘Treatment as usual’ as the control condition and ‘EAT’ as the intervention condition are delivered by radiotherapy dietitians employed at participating hospitals. Nutritional status is assessed using the Patient Generated Subjective Global Assessment (PG-SGA, [10]). The PG-SGA is considered the gold standard measure in oncology nutrition. Assessments are conducted at four time points: during the first week of radiotherapy, last week of radiotherapy, four weeks post radiotherapy and 12 weeks post radiotherapy.

In accordance with the translational nature of our study, ‘treatment as usual’ is defined as the routine dietetic care provided to patients at each of the participating sites. We expect that this care will be guided by national guidelines [11], and as such, should include dietary assessment, counselling and intervention designed to prevent or minimise weight loss during radiotherapy. EAT is grounded in BCC and is designed to encourage HNC patients to maintain sufficient daily nutritional intake despite a range of significant barriers experienced by these patients. These barriers include treatment side effects (pain, oral disfigurement, mucositis, nausea, reduced or no saliva, taste changes and severe loss of appetite) as well as premorbid complications (including tumour-related dysphagia, alcohol misuse and mental illness, for example, depression and poor self-care).

While EAT skills and principles are manualised, EAT is not a linearly structured intervention. Rather, it is based on motivational and behavioural principles such that it can be flexibly integrated by dietitians as part of routine consultations. This is consistent with recommendations for balancing fidelity with the flexibility required when delivering interventions within clinical practice [3]. In light of this, and given that treatment as usual is not manualised (and may vary according to clinician and site), a detailed process evaluation [12] is critical to the evaluation, interpretation and/or dissemination of the EAT intervention. Accordingly, in this paper we describe the EAT fidelity strategies, guided by the NIH treatment fidelity recommendations and refinement offered by Gearing et al. [5].

The EAT project is funded by a Project Grant from the National Health and Medical Research Council [NHMRC; APP1021018; 2011/3654]. The contents of the published material are solely the responsibility of the authors and do not reflect the views of the NHMRC. This study was conducted in accordance with the ethical standards (3.10, 8.01, 8.02 and 8.06) of the American Psychological Association. Informed consent is obtained from all participants. The study protocol has received approval from the Human Research Ethics Committee (HREC) of Hunter New England Health [HREC/12/HNE/108; HNEHREC: 12/04/18/4.06]. Approval has also been received from the following committees: Central Adelaide Local Health Network [HREC/13/RAH/75; SSA/13/RAH/102]; Peter MacCallum Cancer Centre Ethics [SSA/13/PMCC/19]; Western Sydney Local Health District Research Governance [SSA/13/WMEAD/110]; Sir Charles Gairdner Group HREC [2012–136]; Metro South Hospital and Health Service [SSA/13/QPAH/240 and SSA/13/QPAH/241].

### Procedures

This protocol is reported according to four of the five fidelity components proposed by the NIH (Table 1). As per Gearing et al. [5], patient enactment is defined according to treatment efficacy/outcome rather than fidelity and is not discussed in detail. The first four elements are discussed in turn below.

### Study design

#### Ensure the intervention has sound theoretical underpinnings

Development of the EAT intervention was guided by two behaviour change theories ([13, 14]). These theories informed the skills and strategies addressed during training, thereby guiding understanding of the proposed ‘active’ components of therapy. According to the social cognitive theory of self-regulation [13], self-monitoring is central to behaviour change, with the likelihood of a given behaviour influenced by factors such as achievability, reinforcement and accountability [13]. According to the transtheoretical model of change [14], the actual process of behaviour change can be conceptualised in stages (from pre-contemplation through to action and maintenance) [14]. Motivational interviewing (MI) is a complementary clinical method that can enhance personal motivation for change [15]. MI posits that elements of the therapeutic encounter (including empathy, collaboration, exploring ambivalence and eliciting reasons for change) are critical for strengthening commitment, thereby promoting momentum toward behaviour change/maintenance [16].

These theoretical approaches underpin BCC — a patient-centred intervention that incorporates both behavioural and motivational strategies. The EAT intervention is informed by BCC. Specifically, dietitians have been trained in using a written nutrition planner (behavioural contract) to translate patient nutritional requirements into realistic, concrete behavioural goals (achievability). Patients are encouraged to self-monitor their nutrition by ticking each behavioural goal as it is completed (reinforcement). Furthermore, patients are advised that the planner will be reviewed during each session, and a copy kept in the client file for review by the treatment team (accountability). Dietitians have also been trained in using key interpersonal (rapport, respect, collaboration) and communication skills (open questions, reflections, summaries) to elicit ongoing patient feedback regarding the nutritional changes required during radiotherapy. These skills are used to build and consolidate patient motivation and underpin completion of the nutrition planner.

#### Measure treatment dose and intensity

According to best practice guidelines [11], dietetic consultations should occur weekly during radiotherapy, fortnightly for the first six weeks post radiotherapy and then ‘as needed’ thereafter. Given that EAT is designed to be flexibly incorporated into routine dietetic consultations, we used these guidelines in part to inform recommended dose. However, the guidelines do not inform recommended session duration. It is also uncertain how much exposure to EAT is required to promote behaviour change. Therefore, rather than concretely defining the dose and intensity of the intervention (which is also impractical within our translational approach), we are collecting data on the number, frequency and duration of dietetic consultations (across control and intervention conditions). Specifically, the date(s) of all dietetic consultations attended are documented via a chart review. Dietitians are also audio recording all dietetic consultations (from which session duration can be ascertained) and completing a monitoring log for all sessions, irrespective of whether they are audio recorded.

#### Monitor and minimise contamination within and between treatment arms

We have adopted a stepped wedge randomised controlled design. This means that all dietitians began in the ‘control’ arm, offering treatment as usual. Contamination has been minimised as dietitians remained blind to intervention content during the control period. At a randomly allocated timepoint we travelled to each site to provide training in the intervention, after which the intervention phase commenced. Once trained, dietitians were asked to refrain from discussing details of the intervention beyond their site. However, information regarding potential contamination effects will also be apparent via objective, independent rating of session tapes (see discussion under the Treatment Delivery section).

#### Plan for potential setbacks

The current trial is being conducted across multiple sites over approximately two and a half years. Accordingly, several strategies were developed to account for potential attrition of providers. Training was offered to several dietitians at each site. Additional providers were trained such that skilled providers are available to offer cover in the event of dietitian leave and/or turnover. Supplementary training contingencies include repeating the training in person when we returned to the site at the time of the booster session, or offering the workshop via video link. To accommodate busy practice schedules and/or staffing constraints, a condensed version of training has also been developed. Information is being collected about staff turnover across the duration of the trial.

### Training of intervention providers

It is important to ensure that observed intervention differences are not a product of systematic differences in training delivery or clinician skill. This is a particularly important consideration for the current trial, given that training is conducted across multiple sites, with existing clinicians, in a stepwise fashion across several months. Key strategies to minimise the impact of trial duration and provider differences on intervention delivery are summarised below.

#### Standardise training and accommodate provider differences

To maximise the consistency of training, thereby ensuring that dietitians have equivalent training experiences (irrespective of when they join the trial), the following strategies have been applied. All providers were offered a two-day, 12-hour workshop. Workshops were delivered by the same trainers (authors 1–3). All trainers have intimate knowledge of the intervention, as they have worked together to pilot and refine the intervention. A PowerPoint presentation (containing an overview of the background, rationale and core skills of EAT) was used to guide training at all hospital sites. In accordance with recommendations [2], while these core training concepts have been consistent across sites, to accommodate for provider differences, training adequacy has been enhanced by using role plays and discussion to individualise the training to the skill and knowledge level of staff.

Traditional methods for minimising provider differences, such as expecting providers to reach a predetermined competency benchmark before involving them in the trial [17], or pre-selecting providers [4], are impractical in the context of the current trial. Therefore, the focus will be on early, intensive, real-time support and ongoing monitoring and feedback. Accordingly, we accompanied each dietitian during usual practice on the day after training to oversee the implementation of training concepts into actual dietetic consultations. Ongoing supervision and coaching has also been adopted and the frequency, duration and content of each session recorded.

#### Ensure provider skill acquisition

During training, dietitians practiced key skills during role play, and video recordings were made. Role plays focussed on core motivational skills and behavioural strategies. Dietitians had the opportunity to review their video, offer self-reflections and receive individual and/or group feedback (focussing on strengths, areas of development and clinical observations). Skill and knowledge acquisition (adequacy) has been assessed via self-report assessments administered to dietitians before and after the initial workshop, and again after the booster session. Ongoing performance feedback (based on dietitian reflection and supervisor ratings) is offered during supervision. Clinicians are invited to select a session tape they wish to review. If a tape is not nominated by the clinician, one is selected by the supervisor (author 1). The supervisor listens to session tapes and use the Behaviour Change Counselling Index [BECCI; 18] and a study-specific checklist to monitor intervention adherence and competence (these instruments are discussed in detail below under Delivery of Treatment - Reduce differences within treatment and ensure adherence to treatment protocol).

Supervision commenced the week after training and is scheduled to occur fortnightly for at least eight weeks. Intensive preliminary supervision is maintained for approximately two months (that is, until completion of ‘booster’ training, described in the following section). At this time, the frequency of supervision may be reduced pending adequate acquisition of EAT skills. In three separate session tapes, clinicians must demonstrate evidence of ≥80 % of study-specific skills and a mean BECCI score of ≥2.57, an estimate of necessary adherence developed in the piloting of the intervention training. Any concerns regarding clinician delivery of the intervention are discussed with the research team and raised with the clinician. As appropriate, supervisor concerns regarding provider adherence and/or competency may also be raised with the relevant head of dietetics.

#### Minimise ’drift‘ in provider skills

A range of strategies have been adopted to maximise ongoing adherence to the EAT intervention. Key concepts are summarised on supplementary resources (including water bottles, stickers and pocket calendars) and distributed to intervention dietitians at all sites to prompt integration of training concepts into clinical practice. Ongoing supervision and coaching is implemented, including regular review, feedback and discussion of session tapes. We also returned to all sites approximately two months after the initial training to offer a ‘booster’ workshop. Booster training consists of a one-day, 6-hour workshop. This workshop uses a standardised PowerPoint presentation to review key intervention concepts. It is also an opportunity to flexibly respond to learning needs raised by individual clinicians. A combination of discussion, role play, video and feedback is used. Clinicians are also accompanied during their routine consultations to troubleshoot ‘real world’ implementation of the intervention.

### Delivery of treatment

Intervention delivery is the most commonly reported element of fidelity assessment (5). Our use of real world clinicians across a range of settings makes it a particularly important consideration for the current trial.

#### Control for provider differences

As outlined earlier, it is not feasible to use a predetermined criterion to select dietitians for involvement in the trial. However, we have administered a questionnaire prior to training to collect information about prior training and clinical experience of dietitians involved in the trial. We have also monitored provider differences in non-specific treatment factors, namely therapeutic alliance and interpersonal effectiveness. Dietitian and patient perception of therapeutic alliance is assessed at each assessment interval using the Agnew Relationship Measure — Five Item Version — Patient Rated [18]. This five item instrument has been developed as a mechanism for assessing therapeutic alliance within busy clinical settings [18]. It comprises a single ‘core alliance’ domain (consisting of items from the Agnew Relationship Measure bond, partnership and confidence domains; see [9] for further details). Assessment of dietitian interpersonal skills (that is, ‘interpersonal effectiveness’; Cognitive Therapy Scale-Revised; CTS-R; [19]) is derived from blinded ratings of a sample of session audio recordings (this instrument is described in the following section).

#### Reduce differences within treatment and ensure adherence to treatment protocol

In accordance with the gold standard for evaluating intervention delivery, all dietetic sessions are audio recorded and a 20 % sample randomly selected for rating [2]. Randomisation is stratified according to study site, intervention phase (control versus intervention) and dietetic interval (session one versus session five versus on radiotherapy versus off radiotherapy). Session one and five have been chosen as strata based on when we expect key elements of the intervention to occur. On and off radiotherapy have been chosen as strata to account for differences in side effects, nutritional needs and session frequency across these treatment phases.

Coding is completed by an independent assessor blind to the schedule of training and intervention content. A 20 % sample of coded tapes is randomly selected and [1] re-coded by the same independent assessor for *intra-rater* reliability and [2] coded by a separate independent assessor for *inter-rater* reliability. To establish consistency prior to ratings and minimise drift across time, assessors received extensive training and monthly supervision. Before rating study tapes, assessors were required to achieve the following competence benchmarks: ‘excellent’ inter-rater reliability for BECCI, and each of the competence and interpersonal effectiveness items (defined as an intra-class correlation coefficient of ≥ .75) and ‘almost perfect’ agreement on each of the study-specific checklist items (defined as a kappa coefficient of ≥ .81; see [20] for a description of ICC and kappa cut-off scores).

Adherence to BCC techniques is assessed using the BECCI [21]. Relative to other standardised fidelity measures (such as the Motivational Interviewing Treatment Integrity Code [22]), BECCI is less complex and requires less training. BECCI has been shown to demonstrate acceptable levels of validity, reliability and responsiveness [21]. Adherence to study-specific techniques is assessed by a checklist developed by the research team. The study-specific checklist will consist of key intervention elements (see Table 2).

**Table 2:** Study-specific fidelity checklist

	Yes	No
Practitioner discusses how eating/nutrition is an integral part of *radiotherapy* treatment		
Practitioner encourages the patient to discuss their reason(s) for undergoing radiotherapy		
Practitioner collaboratively develops a formal, written nutrition plan with the patient		
Practitioner encourages the patient to discuss their progress towards the goals outlined on their written nutrition plan		

Competence is assessed using a modified version of the Cognitive Behaviour Therapy (CBT) competence item taken from the Cognitive Therapy Scale – Revised (CTS-R, [19]). The wording of this item has been modified to reflect competence in BCC skills relative to CBT. We adapted a standardised assessment for competence in CBT due to the paucity of published standardised competence assessments for BCC skills. Assessors also use the ‘interpersonal effectiveness’ item from the CTS-R as an index of interpersonal skill [19]. This item is rated on a scale of zero to six, with higher ratings indicating greater expression of warmth, concern, confidence, genuineness and professionalism.

### Treatment receipt

Treatment receipt focuses on strategies designed to maximise the likelihood that an intervention is received by patients, that is, whether the patient understands and is able to perform treatment-related skills. EAT integrates motivational and behavioural strategies into standard dietetic consultations with a view to improve patient compliance with dietetic advice. Within this context, defining treatment receipt is challenging, since EAT reflects the spirit in which a consultation is conducted, rather than explicit techniques that are taught to patients. Accordingly, to maximise patient receipt of the intervention, our focus is on strengthening the degree and skill with which EAT is delivered (see strategies outlined previously under the section on treatment delivery). Therefore, treatment receipt is indexed by the independent ratings of BCC and intervention specific skills following training versus standard dietetic consultations.

### Trial status

Until the time of submission, 14 dietitians have completed training (92 % Female; Age range = 26–62; *M* = 38, *SD* = 11.45). To date, due to staff turnover, five dietitians (across three sites) have left the trial. Condensed training has been delivered to four replacement dietitians (across two sites), and the workload of the fifth has been distributed amongst the remaining trained dietitians at that site. Due to upcoming leave, one further dietitian will receive condensed training in September 2015 and complete the booster in November 2015. Objective rating of audio recordings is underway and due to finish May 2016. Participant recruitment is ongoing and due to finish November 2015. Data collection is due to finish May 2016.

## Discussion

Treatment fidelity is an important consideration throughout all stages of behaviour change research. The importance of fidelity was recognised by the NIH Behaviour Change Consortium over a decade ago when they released guidelines recommending that comprehensive fidelity assessment become standard in all behaviour change research [2]. However, despite the potential impact of fidelity on the interpretation and replication of treatment effects, there remains a relative paucity in the detail with which fidelity is commonly reported. We sought to bridge this gap by providing a detailed protocol of fidelity measures adopted in the current trial. Application of fidelity recommendations within a real world setting also presents unique challenges. Therefore, the current paper may also represent a working model for addressing fidelity considerations within translational research. Greater transparency in the reporting of behaviour change research represents an important step in improving the progress and quality of behaviour change research [6].

In order to minimise the impact of provider differences on intervention efficacy, methodological rigour may require controlling for differences in the providers involved in the trial. For example, to maximise intervention delivery, prior research has employed clinicians only if they meet some predetermined level of competence (for example, knowledge, skills, experience [17]). However, as we aimed to develop an intervention that could feasibly be incorporated into the routine practice of HNC dietitians, from a translational perspective, we felt it was important to work with existing providers. Accordingly, we focussed on maximising the effectiveness of our training and conducting comprehensive assessment and monitoring of provider characteristics such that we will be able to comment on the degree to which these factors may impact the delivery of EAT. Future trials could consider conducting a more detailed analysis of provider characteristics (such as the Personality Assessment Inventory [23]) to explore the role of provider differences in willingness to integrate training into their practice. This in turn could help inform the development of improved support for clinicians, who may struggle to integrate the skills they have learned into their clinical practice.

To comment on the impact of an intervention, it is also necessary to specify the treatment duration and dose. We used best practice recommendations [11] to specify the ideal frequency of dietetic consultations. However, no similar recommendations are available for session duration. Indeed it was evident from study outset that session duration is likely to vary according to a number of variables including site, clinician, patient and radiotherapy stage. Therefore, from a fidelity perspective, our focus was on comprehensive assessment and monitoring. By using a range of methods to collect information (such as chart review, dietitian monitoring form and objective rating of audio recordings) on several key process variables (session number duration and frequency; adherence; competence; non-specific factors including therapeutic alliance and interpersonal effectiveness), when we evaluate the impact of the EAT intervention on nutritional status we will be able to account for potential differences in the amount and quality of intervention actually received by participants.

As the training in the current trial was delivered in a stepwise fashion across approximately 12 months, it was also important to minimise systematic differences in the delivery of training. However, we also needed to ensure that we maximised opportunities for learning by catering for provider differences — mainly via individualised supervision, coaching and feedback [3]. Due to the flexibility required to cater for provider differences, assessment and monitoring are crucial. We are therefore documenting the amount of support provided (both frequency and duration), together with the focus of feedback and content of supervision and coaching sessions. Accordingly, we will have the capacity to explore whether treatment outcomes are influenced by the frequency, duration or content of this tailored support.

In light of the importance of ongoing feedback, supervision and coaching for ongoing skill acquisition [24], the design of the current trial could have been further strengthened by tapering the supervision offered by the EAT Trial Coordinator into a site-led peer supervision model. Furthermore, although beyond the resources of the current trial, future research could consider recording all training and supervision sessions and employing an independent assessor to rate the degree and competence to which key elements of the intervention were addressed in supervision. This would allow for even further clarity in the event that site-specific differences in treatment outcome emerge. However, it is important that individual researchers consider the relative costs of this method in terms of whether that degree of information will usefully inform interpretation of treatment outcome and/or replication of treatment effects.

In light of the above discussion, objective assessment of audio recordings is paramount to our ability to comment on the degree and competence with which active elements of the intervention were delivered, how this differs from usual practice and non-specific provider differences. Therefore, a key strength of the current trial is the fidelity procedures adopted to maximise the quality of these data. We have used independent assessors, and our primary assessor is blind to study design and intervention content, and we have trained assessors to a high level of inter-rater reliability and implemented ongoing training and practice ratings to uphold inter and intra-rater reliability throughout the trial. We have also randomly sampled the audio recordings according to strata. Together these strategies ensure that we can confidently use the data obtained for hypothesis testing.

Although fidelity recommendations point to the importance of assessing whether the patient performs intervention skills within a real world setting (‘enactment’), we chose not to consider enactment in detail. While behaviour change is required in the context of standard dietetic intervention (that is, patients need to alter the type of nutrition consumed and may need to alter the frequency, form and mode of delivery), the actual EAT intervention does not teach patients new skills. Rather, dietitians are trained in motivational and behavioural strategies with a view to enhance patient compliance with the nutrition intervention. Enactment, therefore, can be defined as whether the patient is consuming an adequate number of calories, which is indexed by our primary outcome measure: nutritional status. Thus, as has been argued previously (for example, in [5]), treatment enactment is indistinguishable from treatment outcome. This highlights the importance of flexible application of fidelity guidelines: to consider all elements of fidelity, and then focus strategies on those most relevant to the study under consideration.

In summary, attention to fidelity recommendations shaped the development of the intervention and guided the focus and delivery of training, supervision and coaching, together with the objective assessment of dietetic consultations. We have adopted a range of strategies to not only maximise the likelihood that the intervention is delivered as intended, but also to assess the degree and competence with which the proposed active elements of the intervention are present before and after training, as well as non-specific factors known to effect change. Accordingly, we have implemented best practice fidelity recommendations within a translational context such that we can confidently comment on the contribution of the intervention to treatment outcome.

## Trial status

Ongoing (recruiting).

## Abbreviations

BCC: behaviour change counselling
BECCI: Behaviour Change Counselling Index
CBT: Cognitive Behaviour Therapy
EAT: Eating As Treatment
HNC: head and neck cancer
HREC: Human Research Ethics Committee
MI: motivational interviewing
NIH: National Institutes of Health
NMHRC: National Health and Medical Research Council
PG-SGA: Patient Generated Subjective Global Assessment
RCT: randomised controlled trial
